# Strength Characteristics and Micromechanisms of Mucky Soil Co-Stabilized with Geopolymer and Gold Tailings Sand

**DOI:** 10.3390/ma19163554

**Published:** 2026-08-21

**Authors:** Zhaoxia Hu, Lei Yu, Yue Zhao, Biao Luo

**Affiliations:** 1 College of Civil and Construction Engineering, Hunan Institute of Technology, Hengyang 421002, China; 2 College of Urban and Rural Construction, Zhongkai University of Agriculture and Engineering, Guangzhou 510225, China; 3 College of Civil Engineering, Hunan University, Changsha 410082, China; 4 Hunan Engineering Research Center for Disaster Evolution and Reinforcement of Deteriorating Structures, Hunan Institute of Engineering, Xiangtan 411104, China

**Keywords:** gold tailings sand, geopolymer, soil stabilization, mucky soil, strength characteristics, heavy metal leaching

## Abstract

A carbide-slag-activated slag-fly ash geopolymer (CSF) and waste gold tailings sand were used to co-stabilize mucky soil, aiming to promote the valorization of multiple industrial solid wastes and provide a low-carbon treatment approach for mucky soil in river and lake regions. Unconfined compression, direct shear, water stability, scanning electron microscopy coupled with energy-dispersive X-ray spectroscopy (SEM-EDS), and heavy metal leaching tests were conducted to investigate the effects of CSF and gold tailings sand contents on the mechanical properties, water stability, microstructure, and environmental safety of the stabilized soil. The results showed that the unconfined compressive strength (UCS) and shear strength increased with increasing CSF content, whereas the strength gain became marginal when the CSF content exceeded 15%. With the CSF content fixed at 15%, both the strength and water stability initially increased and then decreased as the gold tailings sand content increased. The CSF15-G30 specimen exhibited favorable overall performance, with 7 d and 28 d UCS values of 0.65 and 1.53 MPa, respectively, representing increases of 25.0% and 12.5% relative to CSF15. Its cohesion and internal friction angle reached 88.21 kPa and 47.13°, corresponding to increases of 44.5% and 8.1%, respectively. The water stability coefficients at 7 d and 28 d were 76.9% and 87.6%, respectively. SEM-EDS observations indicated that the cementitious products generated by CSF, together with the filling and skeletal effects of gold tailings sand, enhanced interparticle bonding and matrix densification. Although the concentrations of leached heavy metals increased with increasing gold tailings sand content, all measured values remained below the relevant leaching-toxicity limits. These results demonstrate that an appropriate amount of gold tailings sand can effectively improve the mechanical properties and water stability of CSF-stabilized mucky soil while maintaining satisfactory environmental compatibility.

## 1. Introduction

Large volumes of mucky soil are generated during river and lake dredging, harbor-basin desilting, and underground construction [1,2,3]. Such soils are typically characterized by high natural water content, large void ratio, weak structural integrity, high compressibility, and low bearing capacity, making them unsuitable for direct use as fill materials in road, site-development, and foundation engineering [4,5,6]. Conventional disposal methods, including off-site stockpiling, mechanical dewatering, and excavation and replacement, are not only costly and land intensive, but also increase transportation-related energy consumption and long-term environmental management burdens [7,8]. Chemical stabilization can transform mucky soil from an engineering waste into a reusable fill material by restructuring interparticle connections, refining the pore structure, and enhancing matrix integrity [9,10,11,12]. Although cement and lime are effective stabilizers, their production is associated with high energy consumption and carbon emissions, and relatively high dosages are often required to achieve satisfactory treatment performance in high water content soils [13,14,15,16]. Consequently, the development of low-carbon and efficient binder systems derived from industrial solid wastes has become an important research direction for the beneficial reuse of mucky soil and the synergistic utilization of solid wastes [17,18].

Industrial solid wastes such as fly ash and slag have been widely used for soft-soil stabilization [19,20,21,22,23]. Previous studies have shown that incorporating fly ash or slag into cement-based binders can improve the unconfined compressive strength (UCS), deformation behavior, and microstructure of soft clay and dredged sludge, while partially suppressing the mobility of potentially hazardous elements such as Zn and Ni [24,25,26,27]. Nevertheless, the strength development of such systems remains predominantly governed by cement hydration, and their dependence on conventional carbon-intensive binders has not been fundamentally eliminated. To further increase the utilization of solid wastes, alkali-activated slag–fly ash binders have increasingly been applied to the stabilization of mucky soil, expansive soil, and loess [1,8,28,29,30]. Under alkaline conditions, the reactive aluminosilicate components in slag and fly ash undergo dissolution, depolymerization, and structural reorganization, producing calcium (alumino)silicate hydrate [C-(A)-S-H], sodium aluminosilicate hydrate (N-A-S-H), and hybrid gels that enhance interparticle bonding and reduce pore connectivity. However, these studies have mainly focused on binder-induced chemical cementation, whereas the coupling between such chemical bonding and an intentionally introduced granular waste skeleton remains less understood. Conventional alkali-activated systems generally rely on NaOH and sodium silicate solution, which are associated with relatively high material costs, strong corrosivity, and stringent on-site safety requirements. In the present system, carbide slag acts as a solid alkaline activator and calcium source. Its Ca-rich composition is expressed as CaO in the chemical analysis, while reactive Ca-bearing phases hydrate and/or dissolve in the aqueous environment, generating Ca(OH)_2_ and releasing OH^−^ and Ca^2+^. This promotes the dissolution of reactive Si and Al species from slag and fly ash and facilitates the formation of Ca-rich aluminosilicate gels [31,32,33,34,35]. Carbide slag therefore serves the dual functions of alkaline activation and calcium supplementation, offering a promising substitute for conventional strong-alkali activators while enabling the synergistic utilization of carbide slag, slag, and fly ash.

For high water content mucky soil, reliance solely on cementitious products to bind fine particles may still result in an underdeveloped load-bearing skeleton, nonuniform distribution of reaction products, and insufficient structural stability after water immersion. Previous studies have demonstrated that quartz sand, metakaolin particles, and recycled aggregates can improve the strength and deformation stability of stabilized soft soils through particle-size optimization, pore filling, skeletal support, and interfacial confinement [36,37]. However, conventional granular reinforcement is generally dominated by physical filling, friction, and skeletal support, with relatively limited consideration of the interaction between the granular phase and the cementitious bonding network. Meanwhile, gold mining and beneficiation in China generate substantial quantities of tailings. In 2018 alone, approximately 216 million tonnes of waste gold tailings were produced, accounting for about 17.84% of the total tailings output [38,39,40,41]. Long-term stockpiling not only occupies large areas of land but may also induce dust emissions, leachate migration, and the release of potentially hazardous heavy metals. Owing to crushing and mineral-processing operations, gold tailings commonly occur as fine-sand- or silt-sized particles and are typically rich in siliceous minerals such as quartz [42,43,44,45]. Their irregular morphology, rough surfaces, and fine particle size enable them not only to fill pores and participate in the load-bearing skeleton, but also to provide abundant interfacial sites for the deposition and bridging of geopolymer reaction products. Thus, the proposed “gold tailings sand skeleton–geopolymer bonding–soil-particle interlocking” mechanism involves a coupled physical–chemical reinforcement process rather than conventional granular reinforcement alone [46,47,48]. Nevertheless, the dosage-matching relationship between the geopolymer binder and gold tailings sand has not been systematically established, and it remains unclear how particle filling and interlocking interact with chemical bonding as the tailings content increases. In particular, the transition from beneficial skeletal reinforcement to weakened interfacial bonding at excessive tailings contents, together with its implications for water stability and heavy metal leaching, requires further clarification [49,50,51,52].

Accordingly, an all solid waste-based geopolymer binder was prepared by activating a slag–fly ash blend with carbide slag, and waste gold tailings sand was incorporated as a granular reinforcing material for the physicochemical co-stabilization of mucky soil from river and lake regions. Rather than merely evaluating gold tailings sand as an alternative waste filler, this study focuses on identifying the dosage-dependent coupling between granular skeletal reinforcement and geopolymer-induced chemical bonding. UCS tests were conducted to evaluate the effects of CSF and gold tailings sand contents on the compressive strength and failure characteristics of the stabilized soil. Direct shear tests under different normal stresses were performed to investigate the shear stress–shear displacement response, peak shear strength, and shear strength parameters, while water stability tests were used to assess strength retention after immersion. SEM–EDS characterization was further employed to examine particle morphology, pore features, interfacial bonding, and the elemental composition of representative regions, thereby clarifying how CSF-induced chemical bonding and the filling and skeletal effects of gold tailings sand contribute to the stabilization of the soil. Heavy metal leaching tests were also conducted to assess the environmental safety of geopolymer–gold tailings sand co-stabilized mucky soil (GTSM). The study therefore establishes the relationship among binder–tailings dosage matching, interfacial bonding, granular skeletal reinforcement, macroscopic performance, and environmental compatibility, providing a more mechanistic basis for the synergistic utilization of multiple solid wastes in mucky-soil stabilization.

## 2. Materials and Methods

### 2.1. Materials

**Figure 1:**
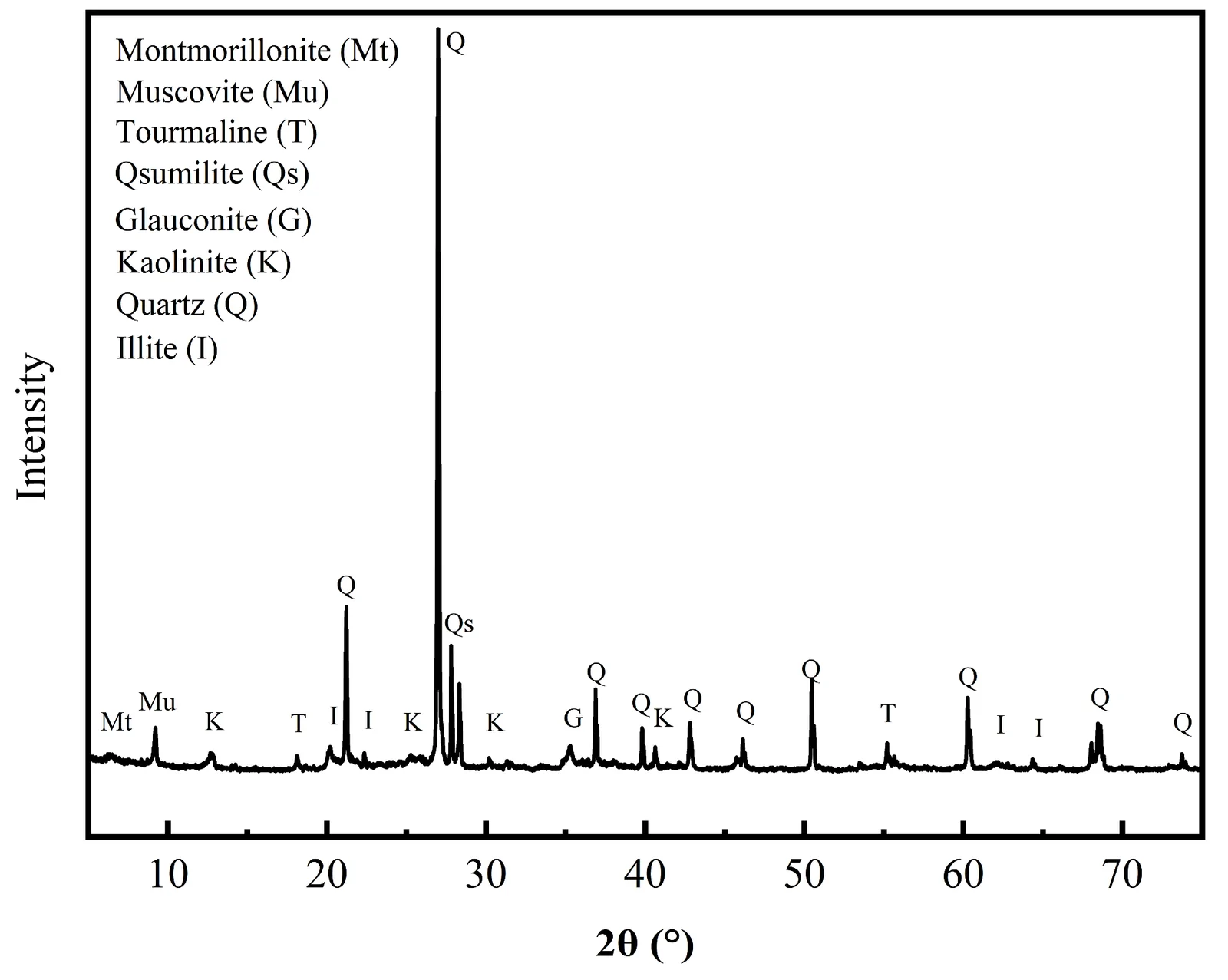
X-ray diffraction (XRD) pattern of the dried mucky soil.

The mucky soil used in this study was collected from an engineering site in Hunan Province, China. Its basic physical properties were determined through laboratory tests, as summarized in Table 1. The collected soil was sequentially oven-dried, crushed, cleared of impurities, and passed through a 2 mm sieve. Before specimen preparation, tap water was added to achieve a target water content of 54.5%. The mixture was thoroughly blended and then sealed for moisture equilibration to prepare the remolded mucky soil. The major chemical composition of the soil is presented in Table 2. As shown in Figure 1, X-ray diffraction (XRD) analysis indicated that quartz, plagioclase, kaolinite, and mica are the main crystalline phases identified in the mucky soil. As shown in Table 2, SiO_2_ and Al_2_O_3_ are the dominant chemical components, with a combined mass fraction of 79.57%.

The binder materials comprised commercially available fly ash, slag, and carbide slag, whose major chemical compositions are also listed in Table 2. The slag and fly ash are rich in SiO_2_ and Al_2_O_3_ and therefore serve as aluminosilicate precursors for the cementitious reactions. Carbide slag mainly consists of Ca(OH)_2_ and can provide both an alkaline reaction environment and a Ca source, thereby promoting the dissolution and reaction of reactive Si and Al species in the slag and fly ash and facilitating the formation of cementitious products [53,54,55].

**Figure 2:**
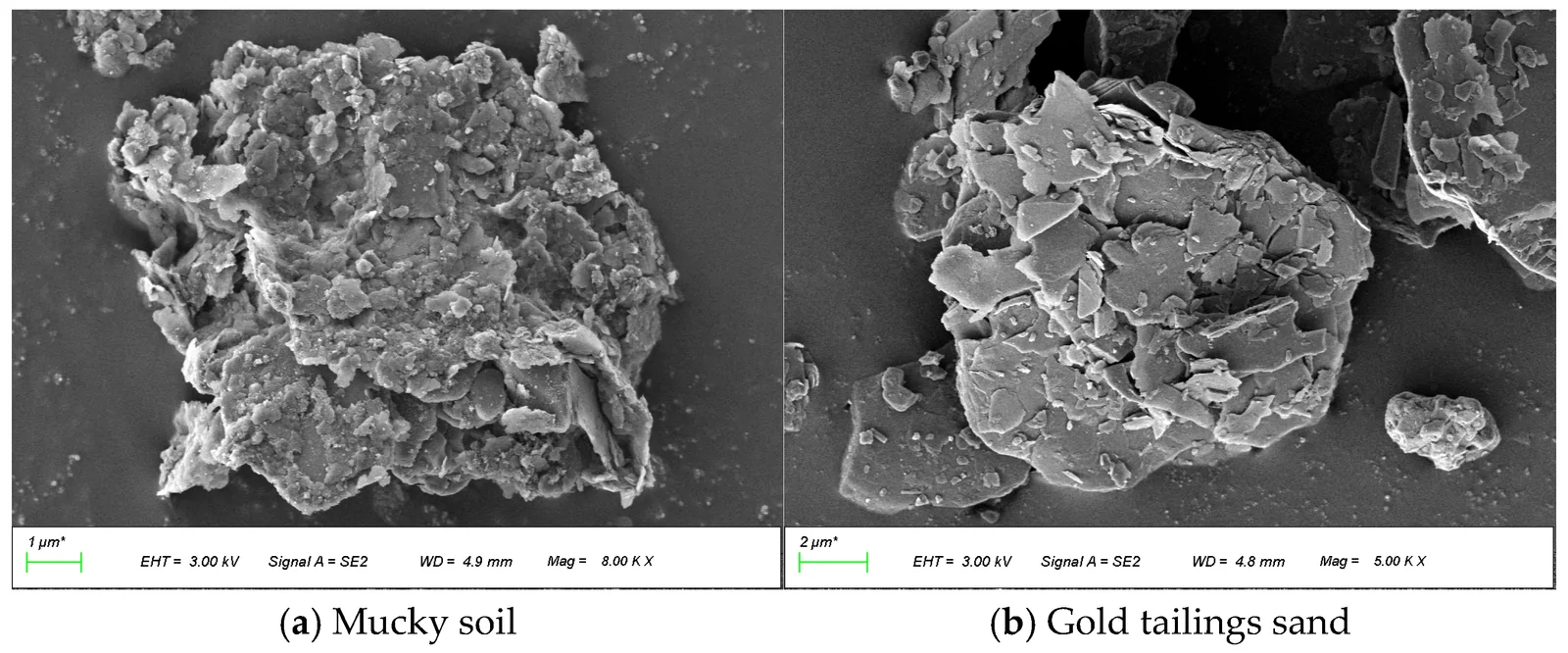
SEM images of the mucky soil and gold tailings sand.

Waste gold tailings sand was employed as a granular reinforcing material to improve the particle-size distribution and mechanical properties of the mucky soil while promoting the beneficial utilization of industrial solid waste. The gold tailings sand was obtained from a gold mine in Hunan Province and was generated during the flotation process. Its median particle size, *D*_50_, was 15.50 μm. The microstructures of the mucky soil and gold tailings sand are shown in Figure 2. The gold tailings particles are predominantly platy, blocky, and irregularly angular, with relatively rough surfaces that favor particle interlocking and the attachment of cementitious products. As shown in Table 2, the mass fractions of SiO_2_, Al_2_O_3_, and CaO in the gold tailings sand are 66.43%, 10.10%, and 6.56%, respectively. Owing to the composition of the parent ore and the mineral-processing procedure, the gold tailings sand also contains potentially hazardous heavy metals. The measured concentrations of Cd, As, Zn, Pb, and Cu are 0.79, 230, 168, 12.0, and 359 mg/kg, respectively. Therefore, leaching tests were further conducted to evaluate the environmental safety of its use in stabilized soil.

### 2.2. Mix Design and Specimen Preparation

Preliminary laboratory screening tests were conducted to determine an appropriate carbide slag–slag–fly ash binder composition based on strength development and mixture workability. Accordingly, the mass ratio of carbide slag, slag, and fly ash was fixed at 40:50:10. Based on this binder composition, eight mix proportions were designed (Table 3) to investigate the effects of CSF and gold tailings sand contents on the stabilization performance of mucky soil. For all mixtures, the dry mass of mucky soil was fixed at 100 g, and both the CSF content and gold tailings sand content were defined as their respective mass ratios to the dry mucky soil. The CSF contents were set at 5%, 10%, 15%, and 20%, while the gold tailings sand contents were varied from 0% to 40%. The mixture was prepared using a two-step procedure in which the soil–tailings phase and the CSF binder were independently water-controlled. The equivalent water content of the combined dry mucky soil and gold tailings sand was maintained at 54.5%, while the water-to-binder ratio of CSF was fixed at 0.30. For clarity, the soil stabilized solely with CSF is referred to as geopolymer-stabilized mucky soil (GSM), whereas that co-stabilized with CSF and gold tailings sand is referred to as GTSM. The corresponding specimens were designated CSF5, CSF10, CSF15, CSF20, and CSF15-G10 to CSF15-G40, where, for example, CSF15-G10 denotes a specimen containing CSF and gold tailings sand at 15% and 10% of the dry mucky soil mass, respectively.

During the first step, the amount of water required to maintain a water content of 54.5% for the combined soil–tailings solids was calculated. Because the gold tailings sand was added later in the dry state, the water corresponding to its dry mass was pre-added to the dry mucky soil during remolding. The remolded soil was then sealed for 24 h to ensure uniform moisture distribution. In the second step, the carbide slag, slag, and fly ash were premixed at a mass ratio of 40:50:10 and mixed with water at a fixed water-to-binder ratio of 0.30 to prepare the CSF binder. The prescribed dry gold tailings sand and freshly prepared CSF were subsequently added to the remolded soil and thoroughly mixed. Cylindrical specimens with a diameter of 50 mm and a height of 100 mm were prepared for the UCS and water stability tests, whereas direct shear specimens were formed in shear rings with a diameter of 61.8 mm and a height of 20 mm. Following the specimen-preparation procedures reported by Luo et al. [2,4], the fresh mixtures were placed and compacted in layers, with each layer lightly scarified to improve interlayer bonding. Demoulding was performed after specimen forming and initial stabilization following the same procedure. After demoulding, the specimens were sealed with plastic film to minimize moisture loss and cured at 20 ± 2 °C and 95 ± 5% relative humidity for 7 and 28 d before testing [56,57,58,59].

### 2.3. Test Methods

UCS and quick direct shear tests were conducted on the prepared specimens. The UCS tests were performed using a CMT5105 universal testing machine (MTS Systems (China) Co., Ltd., Shenzhen, China) at a loading rate of 1 mm/min [2]. For each mix proportion, three replicate specimens were tested, and the mean value was reported as the UCS. During the direct shear tests, normal stresses of 100, 200, 300, and 400 kPa were applied, and the shear rate was set to 0.8 mm/min. The shear strength was determined from the peak shear stress, while the cohesion and internal friction angle were obtained by fitting the Mohr–Coulomb strength criterion.

Water immersion tests were performed to evaluate the water stability of the stabilized soil. After curing, the specimens were completely immersed in distilled water for 24 h and then subjected to UCS testing after removal of surface water. The resistance to water-induced softening was characterized using the post-immersion UCS and the strength retention ratio before and after immersion. Representative specimens were selected for SEM–EDS characterization. Samples were collected from relatively undisturbed internal regions, freeze-dried, trimmed, and gold-coated before observation. Particle morphology, pore characteristics, and interfacial bonding were examined using a TESCAN MIRA LMS scanning electron microscope (TESCAN, Brno, Czech Republic), while the elemental composition and spatial distribution in representative regions were analyzed by EDS.

Heavy metal leaching tests were conducted using an acetic acid buffer solution prepared from analytical-grade glacial acetic acid. The cured specimens were crushed and passed through a 9 mm sieve, and extraction was performed at a liquid-to-solid ratio of 20:1 L/kg at 23 ± 2 °C and 30 ± 2 r/min for 18 ± 2 h. The leachate was filtered through a 0.6–0.8 μm membrane and analyzed for Cd, As, Zn, Pb, and Cu using an Agilent 7850 inductively coupled plasma mass spectrometer (ICP-MS; Agilent Technologies, Santa Clara, CA, USA). An extraction blank and a spiked recovery sample were included in each batch for quality control.

## 3. Results and Discussion

### 3.1. Unconfined Compressive Strength of Stabilized Mucky Soil

**Figure 3:**
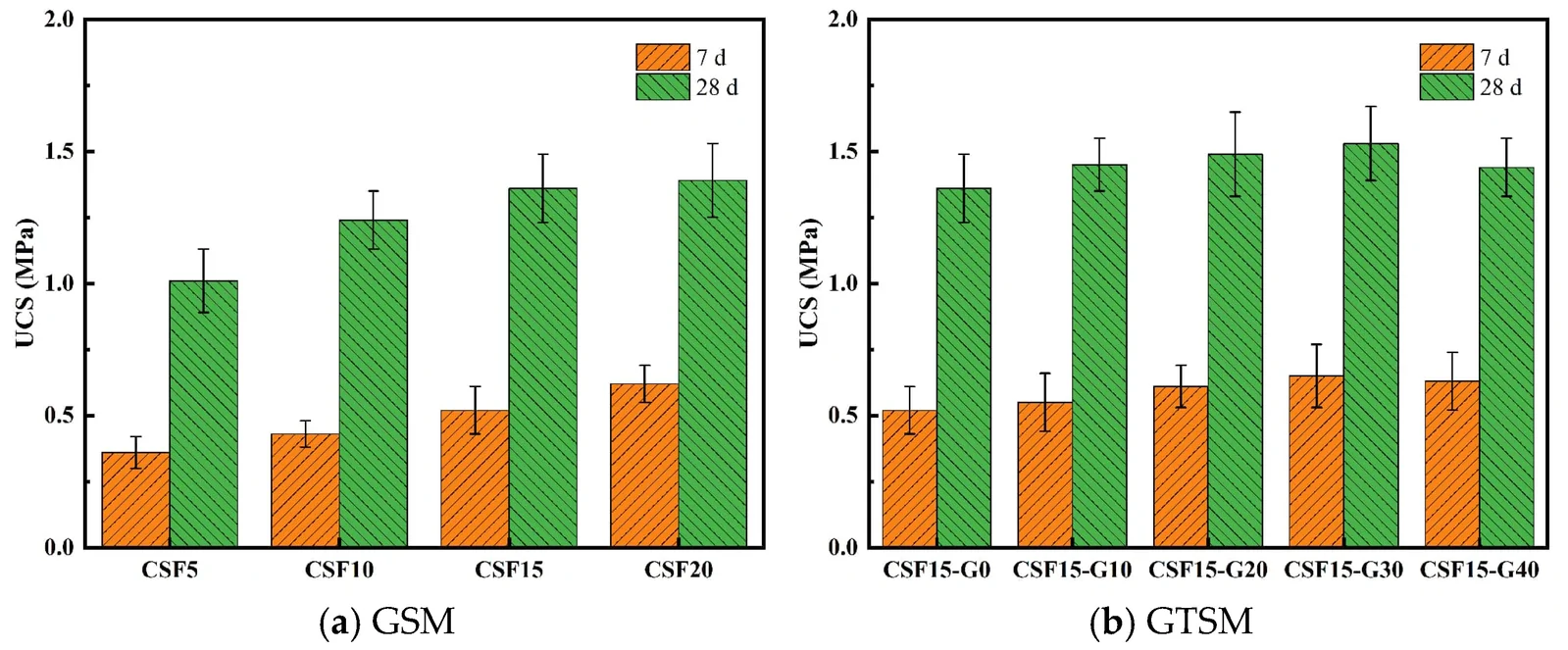
UCS of stabilized mucky soil with different CSF and gold tailings sand contents.

Figure 3 presents the UCS values of GSM and GTSM after 7 and 28 d of curing. As shown in Figure 3a, the UCS of GSM increases continuously with increasing CSF content. When the CSF content increases from 5% to 10%, 15%, and 20%, the 7 d UCS increases from 0.36 MPa to 0.43, 0.52, and 0.62 MPa, corresponding to increases of 19.4%, 44.4%, and 72.2% relative to CSF5, respectively. The corresponding 28 d UCS increases from 1.01 MPa to 1.24, 1.36, and 1.39 MPa, representing increases of 22.8%, 34.7%, and 37.6%, respectively. Increasing the CSF content increases the availability of reactive aluminosilicate species and Ca sources, thereby promoting the formation of cementitious products and strengthening interparticle bonding.

Notably, increasing the CSF content from 15% to 20% raises the 7 d UCS by 19.2%, whereas the corresponding increase in the 28 d UCS is only 2.2%. This finding indicates that a high CSF content primarily promotes early-age strength development, while its contribution to later-age strength becomes progressively limited. Moreover, the relative UCS increase from 7 to 28 d decreases from 180.6% for CSF5 to 124.2% for CSF20, further demonstrating that the strength-development pattern gradually shifts from later-age growth toward early-age hardening as the CSF content increases.

As shown in Figure 3b, at a fixed CSF content of 15%, the UCS of GTSM initially increases and subsequently decreases with increasing gold tailings sand content. Relative to CSF15-G0, the 7 d UCS values of specimens containing 10%, 20%, 30%, and 40% gold tailings sand increase by 5.8%, 17.3%, 25.0%, and 21.2%, respectively, while the corresponding increases at 28 d are 6.6%, 9.6%, 12.5%, and 5.9%. Among the GTSM specimens, CSF15-G30 exhibits the highest UCS, reaching 0.65 MPa at 7 d and 1.53 MPa at 28 d. An appropriate amount of gold tailings sand improves stress transfer through particle filling, interlocking, and skeletal support, while also providing additional surfaces for the deposition of cementitious products and the development of interparticle bonding.

When the gold tailings sand content increases further from 30% to 40%, the 7 d and 28 d UCS values decrease from 0.65 and 1.53 MPa to 0.63 and 1.44 MPa, corresponding to reductions of 3.1% and 5.9%, respectively. Excessive gold tailings sand increases the number and total area of interfaces requiring cementation, whereas the available cementitious products become insufficient to fully coat and connect all particles, resulting in a greater proportion of weak interfaces. Furthermore, the 28 d UCS of CSF15-G30 is 12.5% and 10.1% higher than those of CSF15 and CSF20, respectively, indicating that an appropriate gold tailings sand content can achieve a higher strength level than increasing the CSF content alone. These results confirm a distinct dosage-matching effect between CSF and gold tailings sand, with the maximum UCS obtained for CSF15-G30 under the present experimental conditions.

### 3.2. Shear Strength Characteristics of Stabilized Mucky Soil

#### 3.2.1. Shear Stress–Shear Displacement Relationships

**Figure 4:**
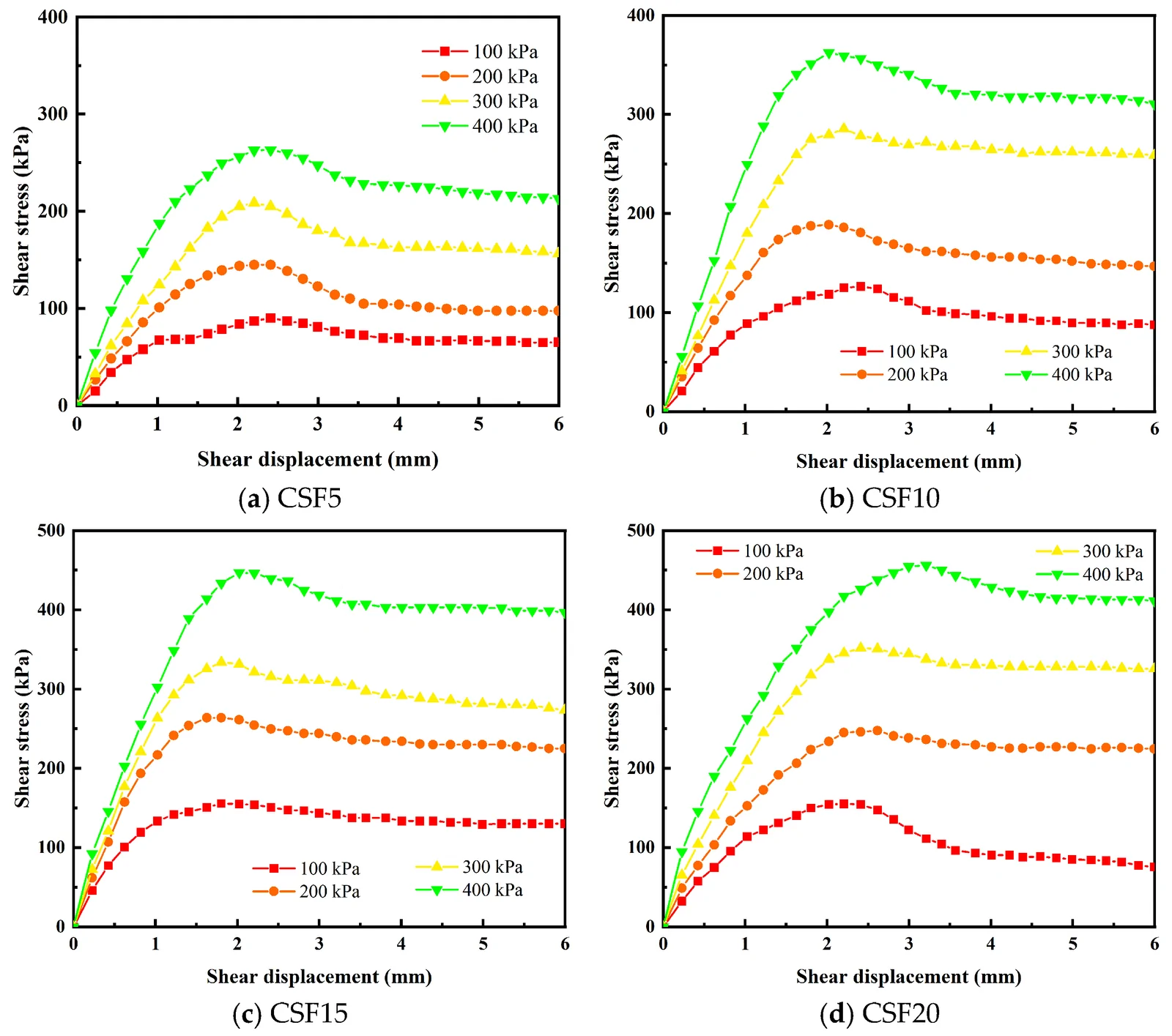
Shear stress–shear displacement curves of GSM with different CSF contents under various normal stresses.

GSM specimens with different CSF contents were cured for 7 d and then subjected to direct shear testing. Figure 4a–d present the representative shear stress–shear displacement curves of GSM under different normal stresses and CSF contents. For all specimens, the shear stress increases rapidly with increasing shear displacement, reaches a distinct peak, and subsequently decreases toward a relatively stable level, indicating pronounced peak-strength and strain-softening behavior. The peak shear stress increases with increasing normal stress, demonstrating that enhanced normal confinement strengthens interparticle contact, friction, and mechanical interlocking, thereby improving the shear resistance of the stabilized soil.

Upon contact with the pore water in the remolded mucky soil, carbide slag provides an alkaline environment that promotes the dissolution and reaction of reactive aluminosilicate species in the slag and fly ash. The resulting cementitious products fill the pores and coat and bridge the soil particles, thereby improving the particle-contact configuration and pore structure and contributing to the relatively high initial stiffness and peak strength of GSM [2]. Beyond the peak stress, progressive breakage of the interparticle bonds and continued development of localized shear bands lead to a gradual reduction in shear stress toward the residual strength.

**Figure 5:**
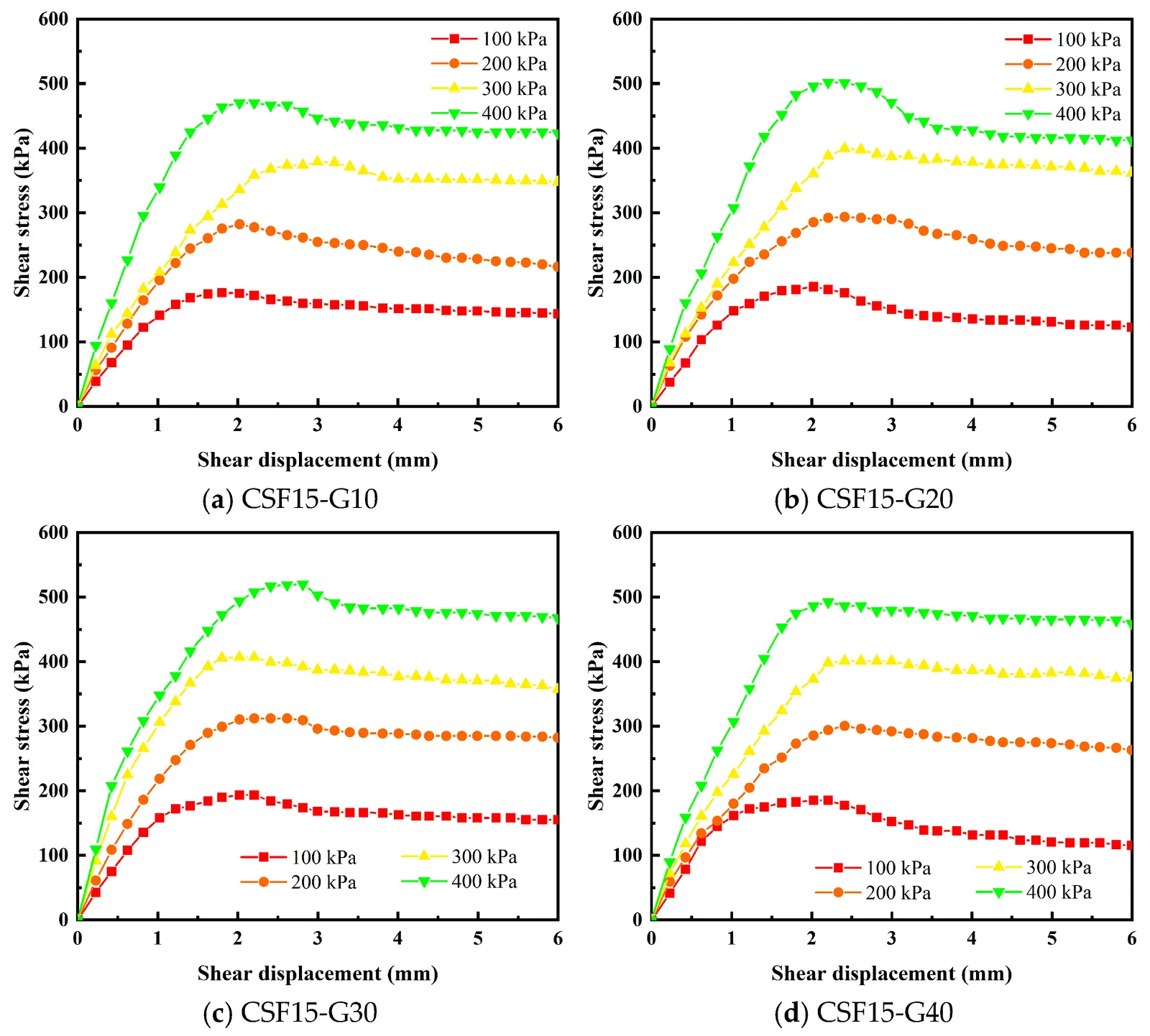
Shear stress–shear displacement curves of GTSM with different gold tailings sand contents under various normal stresses.

Based on the preceding UCS results, CSF15 was selected as the reference mixture to further investigate the effect of gold tailings sand on the shear behavior of the stabilized soil. Figure 5a–d present the representative shear stress–shear displacement curves of GTSM with different gold tailings sand contents under various normal stresses. Similar to GSM, all GTSM specimens exhibit three distinct stages: a rapid increase in shear stress, attainment of the peak value, and subsequent post-peak softening, followed by a gradual transition toward a relatively stable residual strength. The peak shear stress likewise increases with increasing normal stress.

The incorporation of gold tailings sand alters both the particle arrangement and the stress-transfer mechanism within the stabilized soil. An appropriate amount of gold tailings sand enhances shear resistance through particle filling, mechanical interlocking, and skeletal support, while its particle surfaces provide additional interfaces for the deposition of cementitious products and interparticle bonding. Therefore, the shear response of GTSM is governed by the combined effects of CSF-induced chemical bonding and the skeletal reinforcement provided by gold tailings sand. The variations in peak shear strength and shear strength parameters with gold tailings sand content are discussed in the following sections.

#### 3.2.2. Peak Shear Strength

**Figure 6:**
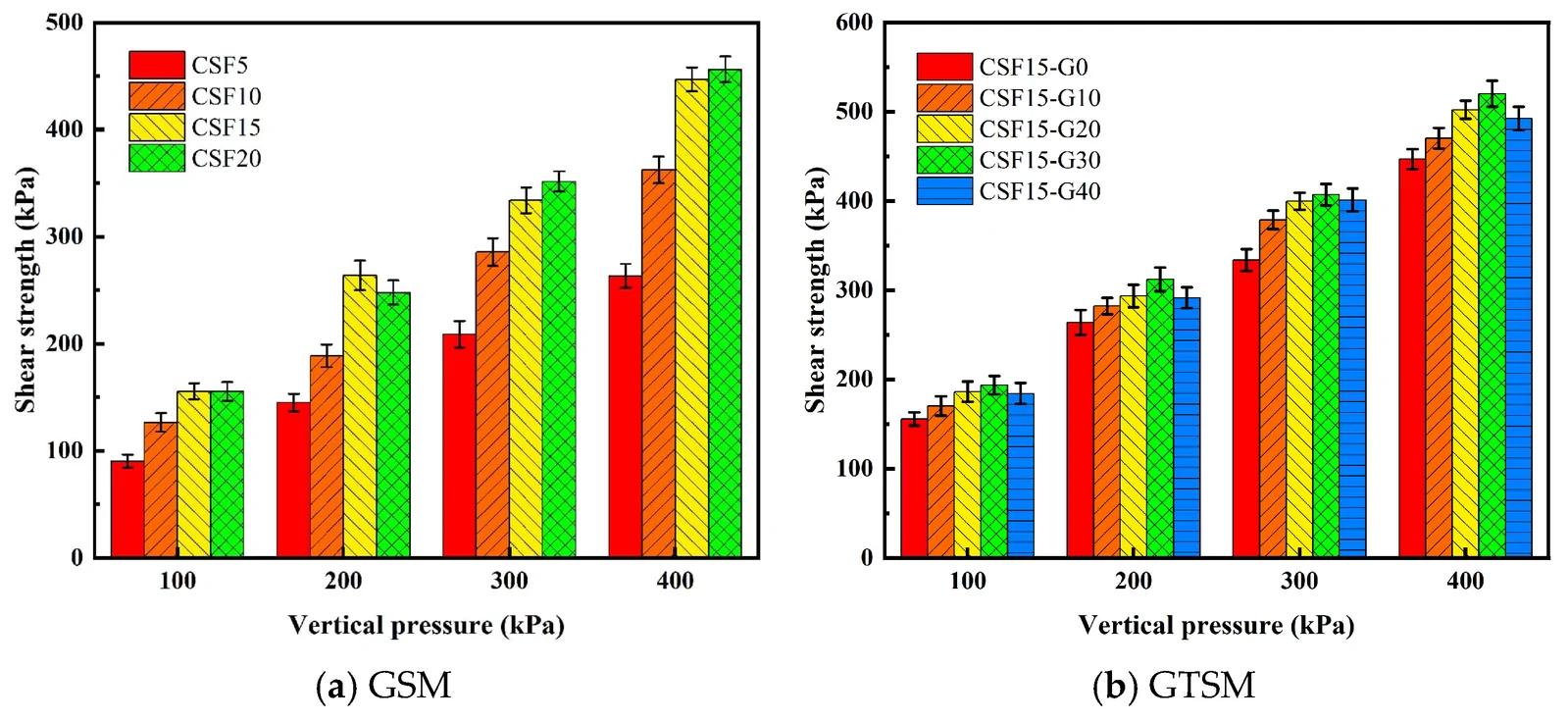
Variation in the shear strength of GSM with CSF content under different normal stresses.

Figure 6a shows the variation in the peak shear strength of GSM with CSF content under different normal stresses. The peak shear strength generally increases with both normal stress and CSF content. Increasing the normal stress enhances interparticle contact and friction and imposes greater confinement on the shear plane, thereby increasing the shear resistance. Under normal stresses of 100, 200, 300, and 400 kPa, the peak shear strengths of CSF15 are 155.6, 263.8, 333.9, and 446.9 kPa, respectively, representing increases of 72.5%, 81.9%, 59.9%, and 69.7% relative to CSF5. As the CSF content increases, a larger quantity of reactive constituents participates in the stabilization reactions, producing additional N-A-S-H and C-(A)-S-H gels that fill the pores and strengthen interparticle bonding, thereby improving the shear resistance of GSM [8,19]. When the CSF content increases from 15% to 20%, however, the corresponding changes in peak shear strength are −0.2%, −6.1%, +5.3%, and +2.1% under the four normal stresses, respectively, indicating no consistent or substantial improvement. This result suggests that the reinforcement effect approaches saturation at a CSF content of approximately 15%, beyond which additional binder does not translate proportionally into further shear-strength gains.

Figure 6b presents the variation in the peak shear strength of GTSM with gold tailings sand content under different normal stresses. The peak shear strength increases continuously as the gold tailings sand content rises from 0% to 30%, but decreases when the content is further increased to 40%. Relative to CSF15-G0, the peak shear strengths of CSF15-G10, CSF15-G20, and CSF15-G30 increase by 9.4–24.5%, 7.0–18.3%, 13.5–21.9%, and 5.2–16.4% under normal stresses of 100, 200, 300, and 400 kPa, respectively. CSF15-G30 exhibits the highest peak shear strength under all normal-stress levels, showing that a gold tailings sand content of 30% provides the most favorable shear performance under the present test conditions. An appropriate amount of gold tailings sand fills the interparticle pores, improves particle packing, and strengthens skeletal support. In addition, the particle surfaces provide favorable sites for the deposition of cementitious products and the development of interparticle bonds, thereby enhancing frictional resistance and stress transfer during shearing.

When the gold tailings sand content increases from 30% to 40%, the peak shear strength decreases by 4.9%, 6.6%, 1.4%, and 5.3% under normal stresses of 100, 200, 300, and 400 kPa, respectively. Excessive fine particles increase the specific surface area and the number of interfaces requiring cementation, thereby reducing the amount of cementitious products available per unit interface. Local agglomeration of fine particles may also introduce weak structural zones, weakening the synergy between skeletal reinforcement and chemical bonding. Consequently, the peak shear strength of GTSM does not increase monotonically with gold tailings sand content, but reaches its maximum for CSF15-G30.

#### 3.2.3. Shear Strength Parameters

**Figure 7:**
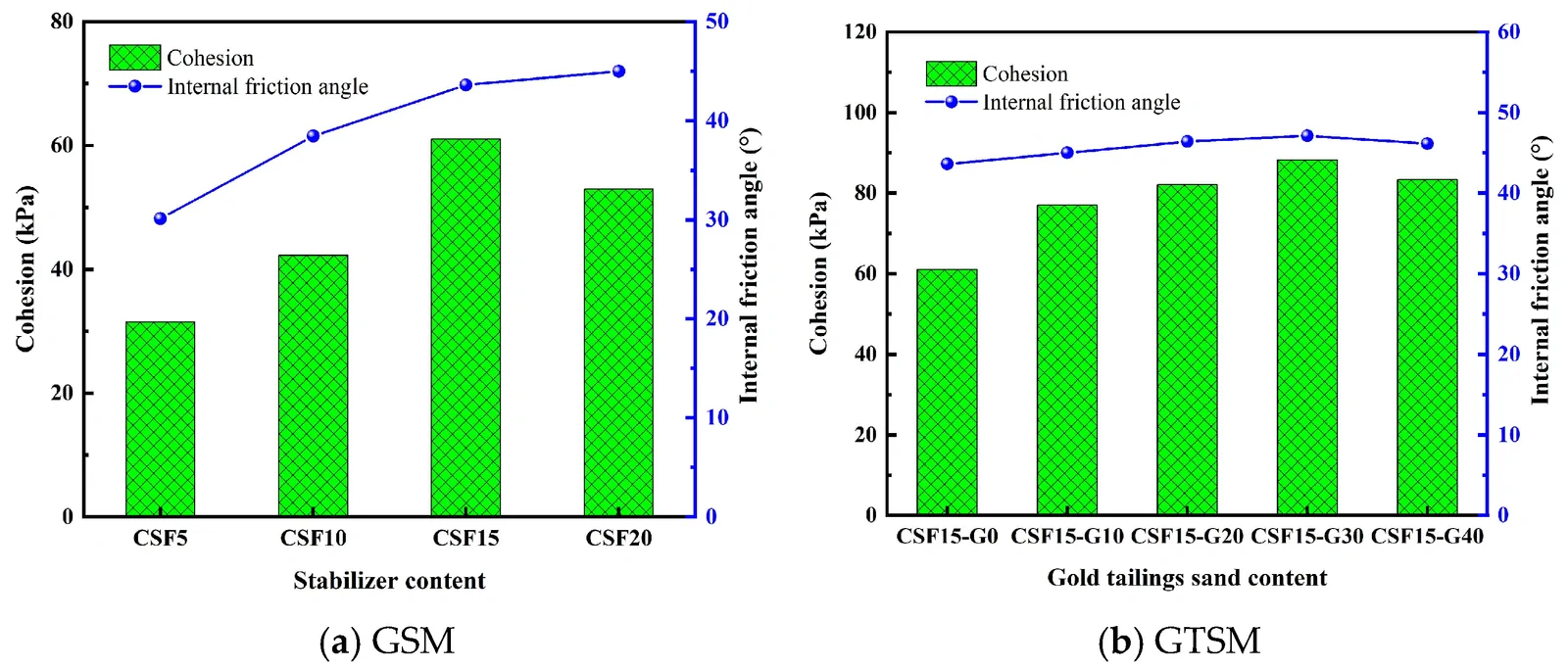
Effects of CSF and gold tailings sand contents on the shear strength parameters of stabilized mucky soil.

Based on the peak shear strengths obtained under four normal stresses, the cohesion *c* and internal friction angle *φ* of GSM were determined by fitting the Mohr–Coulomb strength criterion, as shown in Figure 7a. As the CSF content increased from 5% to 10%, 15%, and 20%, *c* value varied from 31.48 to 42.28, 61.05, and 52.95 kPa, respectively, while *φ* value increased from 30.12° to 38.45°, 43.61°, and 44.99°. Overall, increasing the CSF content improved both shear strength parameters, although their evolution differed. The internal friction angle increased continuously and gradually approached a stable level, whereas the cohesion reached its maximum for CSF15-G0, representing an increase of 93.9% relative to CSF5, and then declined. The N-A-S-H and C-(A)-S-H gels generated by CSF reactions coat and bridge soil particles and fill interparticle pores, thereby enhancing interparticle bonding and mechanical interlocking [5]. When the CSF content increased to 20%, the effective water-to-solids ratio decreased under the constant initial water content. Consequently, part of the binder may have been insufficiently dispersed and reacted, resulting in a nonuniform distribution of cementitious products and the formation of local weak interfaces; therefore, the cohesion did not increase further [2,4].

Figure 7b presents the variations in the shear strength parameters of GTSM with gold tailings sand content. As the gold tailings sand content increased from 0% to 10%, 20%, and 30%, *c* value increased from 61.05 kPa to 77.00, 82.04, and 88.21 kPa, respectively, while *φ* value increased from 43.61° to 45.00°, 46.40°, and 47.13°. Compared with CSF15-G0, the cohesion and internal friction angle of CSF15-G30 increased by 44.5% and 8.1%, respectively, indicating that gold tailings sand contributed more markedly to cohesion enhancement. An appropriate amount of gold tailings sand fills interparticle pores and improves particle packing, while the irregular particles increase frictional resistance and mechanical interlocking. Meanwhile, the deposition of cementitious products at the interfaces between gold tailings sand and soil particles strengthens the overall bonding network [19].

When the gold tailings sand content increased to 40%, *c* and *φ* values decreased to 83.25 kPa and 46.12°, respectively, corresponding to reductions of 5.6% and 2.1% relative to CSF15-G30. Excessive fine particles increase the specific surface area and the number of interfaces requiring cementation, thereby reducing the amount of cementitious products available per unit particle surface. Local particle agglomeration may also generate structurally weak regions. Consequently, the shear strength parameters of GTSM initially increase and then decrease with increasing gold tailings sand content, with the highest values obtained for CSF15-G30.

### 3.3. Water Stability of Stabilized Mucky Soil

**Figure 8:**
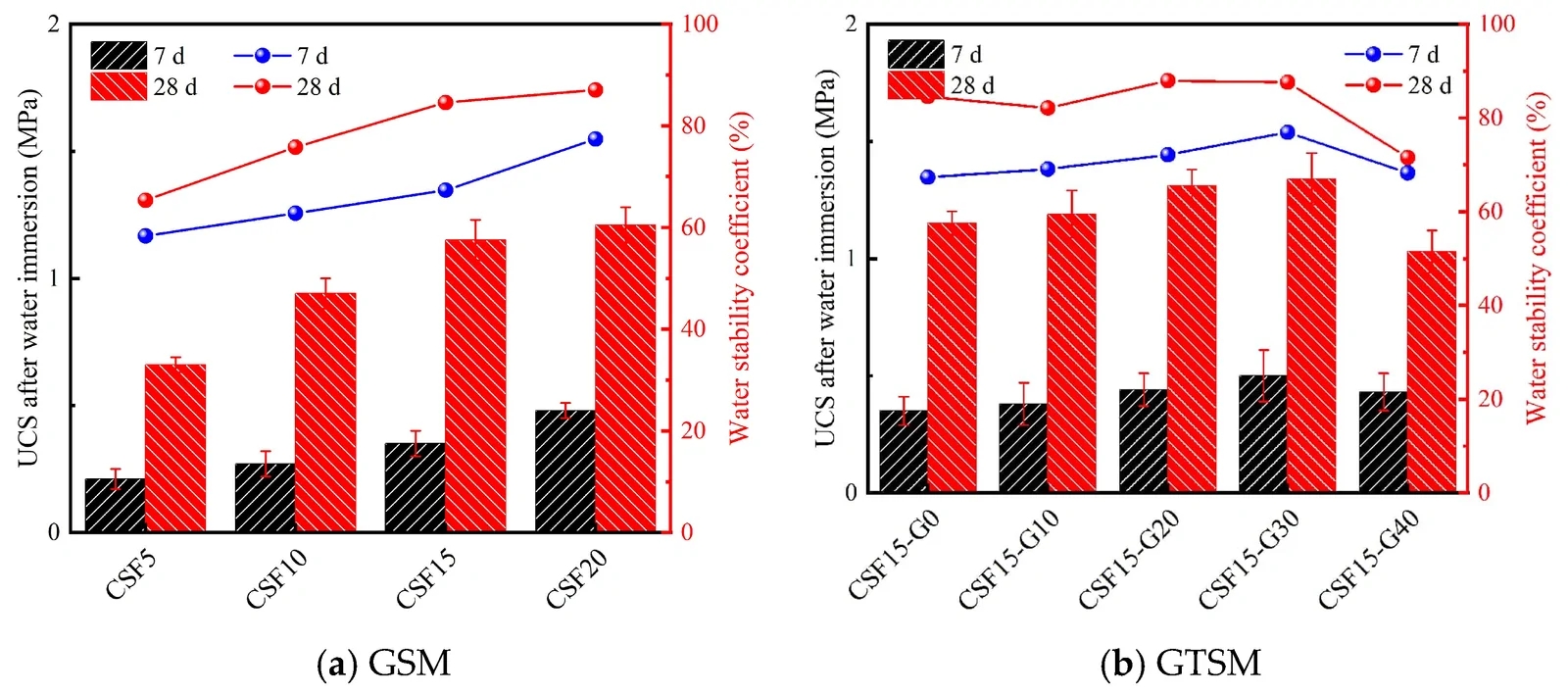
Post-immersion UCS and water stability coefficients of stabilized mucky soil with different CSF and gold tailings sand contents.

The water stability coefficient, *K*, was defined as the ratio of the post-immersion UCS to the UCS of the corresponding unimmersed specimen at the same curing age and was used to characterize strength retention after water immersion. Figure 8a presents the post-immersion UCS and water stability coefficient of GSM with different CSF contents. As the CSF content increased from 5% to 20%, the 7 d post-immersion UCS increased from 0.21 MPa to 0.27, 0.35, and 0.48 MPa, while the corresponding *K* values increased from 58.3% to 62.8%, 67.3%, and 77.4%, respectively. At 28 d, the post-immersion UCS increased from 0.66 MPa to 0.94, 1.15, and 1.21 MPa, and the corresponding *K* values increased from 65.3% to 75.8%, 84.6%, and 87.1%. These results indicate that increasing the CSF content not only enhances the load-bearing capacity after immersion but also mitigates water-induced strength loss. When the CSF content increased from 15% to 20%, the 7 d post-immersion UCS increased by 37.1%, whereas the corresponding increase at 28 d was only 5.2%, suggesting that a higher CSF content contributes more markedly to early-age resistance to water-induced softening. In addition, the *K* values of all specimens increased with curing age, mainly because of continued cementitious reactions, enhanced interparticle bonding, and reduced pore connectivity.

Figure 8b shows the variations in the post-immersion UCS and water stability coefficient of GTSM with gold tailings sand content. At a fixed CSF content of 15%, the post-immersion UCS initially increased and then decreased with increasing gold tailings sand content. Compared with CSF15-G0, the 7 d and 28 d post-immersion UCS values of CSF15-G30 increased from 0.35 and 1.15 MPa to 0.50 and 1.34 MPa, corresponding to increases of 42.9% and 16.5%, respectively. The corresponding *K* values increased from 67.3% and 84.6% to 76.9% and 87.6%. CSF15-G30 exhibited the highest post-immersion UCS, whereas CSF15-G20 showed a slightly higher 28 d water stability coefficient of 87.9%. An appropriate amount of gold tailings sand improves particle packing and skeletal support, while cementitious products formed between the tailings particles and soil particles enhance interfacial bonding and structural stability under immersion conditions. When the gold tailings sand content increased further to 40%, the 7 d and 28 d post-immersion UCS values decreased to 0.43 and 1.03 MPa, representing reductions of 14.0% and 23.1% relative to CSF15-G30, respectively. The corresponding *K* values declined to 68.3% and 71.5%. This result indicates that excessive gold tailings sand markedly weakens resistance to water-induced softening, likely because the increased specific surface area and number of interfaces requiring cementation prevent the limited cementitious products from forming a continuous and stable bonding network.

### 3.4. Micromechanism of Stabilized Mucky Soil

#### 3.4.1. Microstructural Morphology

**Figure 9:**
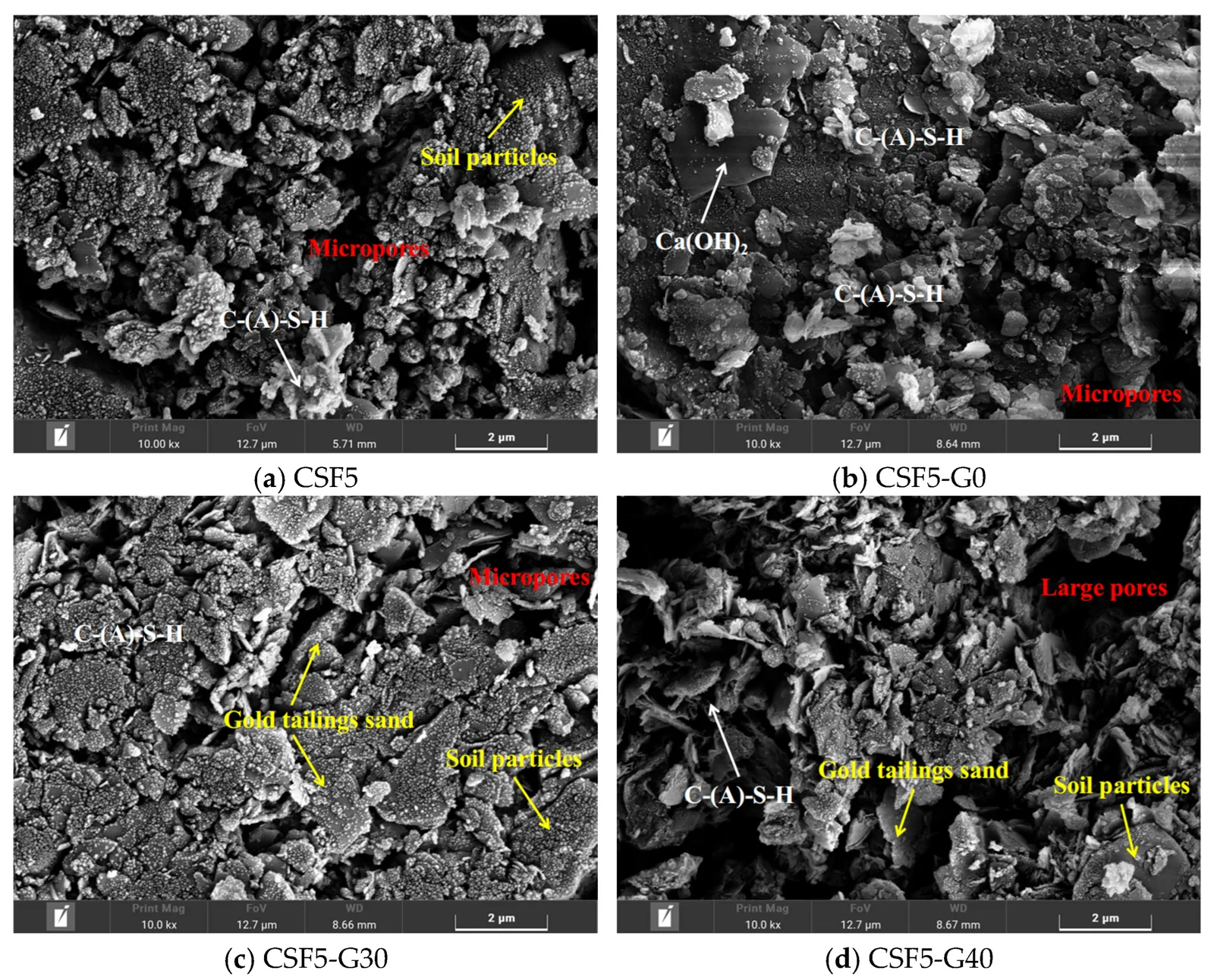
SEM images of representative stabilized mucky soil specimens after 28 d of curing.

Figure 9 presents the SEM images of CSF5, CSF15-G0, CSF15-G30, and CSF15-G40 after 28 d of curing. As shown in Figure 9a, the addition of 5% CSF induces partial aggregation of the soil particles through cementitious bonding; however, numerous micropores remain between the particles. This observation indicates that the limited amount of cementitious products generated at a low CSF content is insufficient to continuously coat and bridge the soil particles, consistent with the relatively low UCS and water stability of CSF5.

When the CSF content increases to 15%, the internal structure becomes noticeably denser, with abundant flocculent and gel-like products adhering to the particle surfaces and forming continuous filling and bridging structures, as shown in Figure 9b. The gel-like products may be preliminarily identified as Ca-, Si-, and Al-rich C-(A)-S-H, whereas the plate-like crystals are likely to be residual Ca(OH)_2_. The specific compositions are further examined using EDS. The hydration of carbide slag and the dissolution–polymerization reactions of reactive aluminosilicate species in slag and fly ash can be simplified as follows [60,61,62,63,64]:

(1) $$CaO+H_{2}O\to Ca(OH{)}_{2}$$

(2) $$\mathrm{Ca}(\mathrm{OH}{)}_{2}⇌{\mathrm{Ca}}^{2+}+2{\mathrm{OH}}^{-}$$

(3) $${\mathrm{Ca}}^{2+}+\mathrm{reactive}\;\mathrm{silicate}\;\mathrm{species}+\mathrm{reactive}\;\mathrm{aluminate}\;\mathrm{species}+H_{2}O\to C\text{-}(A)\text{-}S\text{-}H\;\mathrm{gel}$$

The alkaline environment provided by Ca(OH)_2_ promotes the depolymerization of aluminosilicate networks, while the released reactive Si and Al species subsequently react with Ca^2+^ to form C-(A)-S-H gels. Reactive aluminosilicate components originating from the soil minerals may also participate in these reactions, thereby strengthening interparticle bonding and refining the pore structure [5]. In Ca-rich alkali-activated slag–fly ash systems, C-(A)-S-H-type gels generally constitute the dominant cementitious products.

As shown in Figure 9c, fine gold tailings sand particles are distributed among the soil particles in CSF15-G30 and are coated and bridged by cementitious products, resulting in a denser particle arrangement. Gold tailings sand not only fills relatively large pores but also acts as a rigid microaggregate within the load-bearing skeleton. Meanwhile, the cementitious products formed at the tailings sand–soil interfaces enhance stress transfer between the particles. These microstructural features, including particle filling, skeletal support, and interfacial bonding, are consistent with the relatively high UCS, shear strength, and post-immersion strength of CSF15-G30.

When the gold tailings sand content increases to 40%, pronounced macropores and loosely packed regions appear within the matrix, as shown in Figure 9d. Because the CSF content remains unchanged, the excessive addition of gold tailings sand increases the total particle surface area requiring cementation. Consequently, the available cementitious products become insufficient to fully coat and connect all particles, leading to weak contacts between some tailings and soil particles and a reduction in structural continuity. This microstructural feature accounts for the lower UCS, shear strength, and water stability of CSF15-G40 compared with CSF15-G30.

#### 3.4.2. Energy-Dispersive X-Ray Spectroscopy Analysis

Table 4 presents the average SEM–EDS elemental compositions of representative cementitious regions in CSF5, CSF15-G0, CSF15-G30, and CSF15-G40. O, Si, Al, and Ca are the dominant elements in all specimens, whereas the Na content remains relatively low, ranging from 0.64% to 0.86%. This elemental composition indicates that the stabilization reactions are dominated by Ca-rich aluminosilicate products. Combined with the gel-like morphology described above and the coexistence of Ca, Si, and Al, the cementitious regions are inferred to contain predominantly C-(A)-S-H-type gels [65,66,67].

In the cementitious region of CSF5, the Ca, Si, and Al contents are 4.35%, 18.52%, and 11.06%, respectively, corresponding to Si/Al and Ca/Si ratios of 1.67 and 0.23. These values indicate limited formation of Ca-bearing reaction products at the low CSF content. When the CSF content increases to 15%, the Ca content in CSF15-G0 rises to 7.33%, representing an increase of 68.5% relative to CSF5, while the Ca/Si ratio increases to 0.38. The corresponding Si and Al contents are 19.05% and 10.28%, respectively. The increased Ca/Si ratio suggests greater participation of Ca in the formation of Ca-rich reaction products commonly associated with C-(A)-S-H-type gels, which may enhance particle coating, pore filling, and interfacial bonding. These microstructural changes are consistent with the observed increase in UCS and other macroscopic mechanical properties [2].

The cementitious region of CSF15-G30 contains 21.13% Si, 9.79% Al, and 6.24% Ca, with Si/Al and Ca/Si ratios of 2.16 and 0.30, respectively. Compared with CSF15-G0, the Si content increases by 10.9%, whereas the Ca content decreases by 14.9%. This shift reflects the stronger contribution of siliceous particles following the incorporation of gold tailings sand, while the interface still exhibits a distinct coexistence of Ca, Si, and Al. Based on the bulk oxide composition of the gold tailings sand, the nominal atomic ratios of Si/Al and Ca/Si are approximately 5.59 and 0.106, respectively. The substantially lower Si/Al and higher Ca/Si ratios measured in the cementitious region of CSF15-G30 therefore indicate that the local composition is not governed solely by the tailings particles, but also by the enrichment of Ca and Al and the deposition of cementitious products. The moderate Si/Al and Ca/Si ratios, together with the filling and skeletal effects of gold tailings sand, are conducive to the development of a relatively continuous interfacial bonding network, thereby improving stress transfer and being consistent with the highest UCS observed for CSF15-G30.

When the gold tailings sand content increases to 40%, the Si content in CSF15-G40 rises to 25.74%, whereas the Ca content decreases to 3.86%. Meanwhile, the Si/Al ratio increases to 2.75 and the Ca/Si ratio decreases to 0.15. The higher Si/Al and lower Ca/Si ratios indicate a more Si-rich and Ca-deficient local composition, which may reflect a reduced availability of Ca-rich reaction products for bonding the increased particle interfaces [2,68,69,70]. Consequently, particle coating and interfacial bridging may become less effective, contributing to weaker stress transfer and a reduction in macroscopic strength.

### 3.5. Heavy Metal Leaching Characteristics

To evaluate the environmental suitability of GTSM as a fill material for foundations or road subgrades, heavy metal leaching tests were conducted on specimens cured for 7 d, and the results are presented in Table 5. The Cd concentrations in all specimens were below the detection limit of 0.01 mg/L and were therefore not quantitatively compared. The leaching concentrations of Zn, As, Cu, and Pb from CSF15 are 0.21, 0.23, 0.38, and 0.11 mg/L, respectively. All four concentrations increase progressively with increasing gold tailings sand content. At a gold tailings sand content of 40%, the corresponding concentrations rise to 1.85, 2.36, 2.68, and 0.27 mg/L, representing increases of approximately 781%, 926%, 605%, and 145% relative to CSF15, respectively. These results indicate that gold tailings sand is the principal source of heavy metal release from the stabilized soil and that its dosage directly affects the leaching levels of potentially hazardous elements.

According to GB 5085.3–2007 [71], the extraction-toxicity limits for Zn, As, Cu, and Pb are 100, 5, 100, and 5 mg/L, respectively. The concentrations measured for CSF15-G40 correspond to 1.85%, 47.2%, 2.68%, and 5.40% of the respective limits, with none exceeding the prescribed threshold. Notably, the As concentration represents a substantially higher proportion of its limit than the other elements, indicating that As should be prioritized in the long-term environmental risk assessment of GTSM. GB 5085.7–2019 [72] establishes the general procedures and decision rules for hazardous-waste identification, whereas the numerical extraction-toxicity limits adopted in this study are specified in GB 5085.3–2007 [71].

Although the incorporation of gold tailings sand increases the leaching concentrations of heavy metals, the C-(A)-S-H-type gels formed through CSF reactions can restrict metal mobility through surface adsorption, physical encapsulation, and ionic binding. Matrix densification also increases the transport path of the extraction solution within the stabilized soil. Therefore, within the curing age and testing scope considered, GTSM exhibits no exceedance of the specified leaching-toxicity limits, supporting the preliminary environmental feasibility of using gold tailings sand for the co-stabilization of mucky soil. Nevertheless, because the As concentration in CSF15-G40 reaches 47.2% of the regulatory limit, its long-term release stability under extended curing, different pH conditions (e.g., acid rain and alkaline environments), and wetting–drying cycles requires further evaluation before large-scale engineering application.

**Table 5:** Heavy metal leaching concentrations of geopolymer–gold tailings sand co-stabilized mucky soil (mg/L).

Heavy Metal	CSF15	CSF15-G10	CSF15-G20	CSF15-G30	CSF15-G40	Regulatory Limit [72]
Zn	0.21	0.53	0.90	1.46	1.85	100
Cd	<0.01	<0.01	<0.01	<0.01	<0.01	1
As	0.23	0.72	1.34	1.95	2.36	5
Cu	0.38	0.87	1.46	2.03	2.68	100
Pb	0.11	0.16	0.17	0.21	0.27	5

## 4. Conclusions

This study investigated the co-stabilization of mucky soil using a carbide-slag-activated slag–fly ash geopolymer and gold tailings sand. UCS, direct shear, water stability, SEM–EDS, and heavy metal leaching tests were performed to evaluate the effects of CSF and gold tailings sand contents on the macroscopic properties and microstructure of the stabilized soil. The main conclusions are as follows:

(1) The UCS of GSM increases with increasing CSF content, although the later-age strength gain gradually diminishes at higher dosages. Increasing the CSF content from 15% to 20% raises the 7 d UCS by 19.2%, but increases the 28 d UCS by only 2.2%, indicating that 15% CSF provides a sufficiently effective stabilization level. With CSF15-G0 as the reference mixture, the UCS of GTSM initially increases and then decreases with increasing gold tailings sand content. CSF15-G30 achieves 7 d and 28 d UCS values of 0.65 and 1.53 MPa, respectively, representing increases of 25.0% and 12.5% relative to CSF15-G0.

(2) Both GSM and GTSM exhibit distinct peak strength and post-peak softening behavior, and their shear strength increases with normal stress. When the CSF content increases from 5% to 15%, the shear strength of GSM under normal stresses of 100–400 kPa increases by 59.9–81.9%, while the cohesion and internal friction angle increase from 31.48 kPa and 30.12° to 61.05 kPa and 43.61°, respectively. The highest shear-strength parameters were obtained at a gold tailings sand content of 30%, with the cohesion and internal friction angle of CSF15-G30 reaching 88.21 kPa and 47.13°, respectively. Further increasing the gold tailings sand content to 40% reduces the shear performance.

(3) Increasing the CSF content improves both the post-immersion UCS and water stability of the stabilized soil. As the CSF content increases from 5% to 20%, the water stability coefficients at 7 d and 28 d increase from 58.3% and 65.3% to 77.4% and 87.1%, respectively. CSF15-G30 exhibits post-immersion UCS values of 0.50 MPa at 7 d and 1.34 MPa at 28 d, with corresponding water stability coefficients of 76.9% and 87.6%. When the gold tailings sand content increases to 40%, the 28 d water stability coefficient decreases to 71.5%, indicating that excessive tailings addition weakens resistance to water-induced softening.

(4) SEM–EDS analysis shows that the C-(A)-S-H-type gels generated by CSF reactions coat and bridge soil particles while filling the pore space. A relatively continuous “gold tailings sand particle–interfacial bonding region–stabilized soil matrix” structure develops in CSF15-G30, in which gold tailings sand provides particle filling and rigid skeletal support, whereas the cementitious products strengthen interfacial bonding. When the gold tailings sand content increases to 40%, the Si content in the analyzed region increases from 21.13% to 25.74%, while the Ca content decreases from 6.24% to 3.86%, reflecting a relative deficiency of Ca-rich cementitious products and reduced interfacial continuity.

(5) The leaching concentrations of Zn, As, Cu, and Pb increase with increasing gold tailings sand content, but remain below the relevant leaching-toxicity limits for all specimens. For CSF15-G40, the corresponding concentrations are 1.85, 2.36, 2.68, and 0.27 mg/L, respectively. This accounts for a comparatively high proportion of its regulatory limit and therefore warrants particular attention in long-term engineering applications. Overall, GTSM demonstrates satisfactory preliminary environmental feasibility under the present test conditions.

Future studies should consider prolonged immersion, wetting–drying cycles, and coupled environmental actions, together with field-scale validation and life-cycle assessment, to facilitate the engineering application of multi-solid-waste co-stabilization technologies.

## Figures and Tables

**Table 1:** Basic physical properties of the mucky soil.

Natural Moisture Content/wt.%	Maximum Dry Density/(g·cm^−3^)	Organic Matter Content/wt.%	Porosity	Relative Density	Liquid Limit/%	Plastic Limit/%
54.5	1.82	1.02	1.56	2.75	26.7	16.5

**Table 2:** Major chemical compositions of the raw materials.

Raw Materials	Component Content/%						
	SiO_2_	Al_2_O_3_	Fe_2_O_3_	MgO	CaO	Na_2_O	LOI
Mucky soil	63.52	16.05	5.469	0.96	0.83	0.50	8.65
Carbide slag	2.63	1.75	0.08	0.22	68.42	0.44	25.13
slag	33.46	16.20	0.79	9.20	40.16	0.18	1.35
Fly ash	45.23	33.63	7.59	0.59	5.37	2.28	3.06
Gold tailings sand	66.43	10.10	3.25	1.24	6.56	0.90	2.15

**Table 3:** Mix proportions and specimen designations.

No.	CSF Content/%	Filler Content/%	Carbide Slag/g	Fly Ash/g	Slag/g	Dry Mucky Soil/g	Gold Tailings Sand/g
CSF5	5	-	2	0.5	2.5	100	-
CSF10	10	-	4	1	5	100	-
CSF15/CSF15-G0	15	-	6	1.5	7.5	100	-
CSF20	20	-	8	2	10	100	-
CSF15-G10	15	10	6	1.5	7.5	100	10
CSF15-G20	15	20	6	1.5	7.5	100	20
CSF15-G30	15	30	6	1.5	7.5	100	30
CSF15-G40	15	40	6	1.5	7.5	100	40

**Table 4:** Average elemental compositions and characteristic atomic ratios of representative cementitious regions in stabilized mucky soil determined by EDS.

No.	Elemental Content (at.%)					Si/Al	Ca/Si
	Na	Ca	Al	Si	O		
CSF5	0.79	4.35	11.06	18.52	53.36	1.67	0.23
CSF15-G0	0.86	7.33	10.28	19.05	50.15	1.85	0.38
CSF15-G30	0.73	6.24	9.79	21.13	54.69	2.16	0.30
CSF15-G40	0.78	3.86	9.35	25.74	57.64	2.75	0.15

## Data Availability

The original contributions presented in this study are included in the article. Further inquiries can be directed to the corresponding author.
